# In Silico Approach Gives Insights into Ig-like Fold Containing Proteins in *Vibrio parahaemolyticus*: A Focus on the Fibrillar Adhesins

**DOI:** 10.3390/toxins14020133

**Published:** 2022-02-10

**Authors:** Dan Wang, Haoran Wang

**Affiliations:** 1 School of Food and Advanced Technology, Massey University, Palmerston North 4410, New Zealand; 2 School of Medicine, Jiangsu University, Zhenjiang 212000, China; wanghr_hi@126.com

**Keywords:** fibrillar adhesin, homologue, protein interface, drug target

## Abstract

Immunoglobulin-like (Ig-like) fold domains are abundant on the surface of bacteria, where they are required for cell-to-cell recognition, adhesion, biofilm formation, and conjugative transfer. Fibrillar adhesins are proteins with Ig-like fold(s) that have filamentous structures at the cell surface, being thinner and more flexible than pili. While the roles of fibrillar adhesins have been proposed in bacteria overall, their characterization in *Vibrio parahaemolyticus* has not been established and, therefore, understanding about fibrillar adhesins remain limited in *V. parahaemolyticus*. This in silico analysis can aid in the systematic identification of Ig-like-folded and fibrillar adhesin-like proteins in *V. parahaemolyticus*, opening new avenues for disease prevention by interfering in microbial interaction between *V. parahaemolyticus* and the host.

## 1. Introduction

*Vibrio parahaemolyticus* is a marine-oriented pathogen, which can be detected in estuarine, coastal environments and seafood. *V. parahaemolyticus* has resulted in significant economic loss in animal production and increased spread of foodborne diseases [1]. Its virulence factors assist in spreading pathogenesis, by being involved in adhesion, effector delivery, motility, exotoxin production, exoenzyme production and biofilm formation of bacteria [2]. The intercellular communication and extracellular biofilm matrix development start with cell adhesion, which relies on the receptors located on cell surfaces. Among these receptors, the immunoglobulin-like [3] fold domains are the most widely distributed and typical class of proteins [4].

Ig-like domains play critical roles in *Vibrio*. VesB is a serine protease in *Vibrio cholerae* with an Ig-like fold domain at C-terminal of the protein; the deletion of the Ig-like domain resulted in the degradation of the protein [5]. The roles of Ig-like fold domains have been proposed to be related with stability, substrate specificity, cell surface association, and type II secretion of extracellular proteins [6]. In *Vibrio fischeri*, the mutant of *Vibrio* Ig-like protein (Vig) was created, and the Ig-like protein was associated with roles in influencing luminescence produced by *V. fischeri* and the ability of the symbiont to colonize the squid [7]. Chitinases, important extracellular enzymes in the marine environment, are widely distributed in Vibrio cells and contain Ig-like domains, implying Ig-like domains in chitin identification and utilization [8]. Ig-fold like domains have been found in double-stranded DNA bacteriophages, the weak, probable non-specific interaction between these domains and the bacterial cell wall could remain the phage gliding or bouncing on the bacteria, suggesting Ig-like fold domain roles of phage infection [9]. Ig-like domains are essential for *Vibrio* phage ØVC8 interactions with the human intestine, and are related to the lytic activity of the phage in preventing *V. cholerae* O1 colonization [10]. Ig-like domains were frequently found among *Vibrio* adhesins which play essential roles in adhesion and invasion of pathogens into host cells [11]. Compared to fimbrial adhesins, fibrillar adhesins are thinner, more flexible, 10 times smaller; and they can be involved in biofilm formation and cell-cell interactions [4]. Although fibrillar adhesin-like proteins play essential roles in adhesion, due to their size and often elaborate multidomain architectures, it is challenging to characterize these polypeptides structurally and functionally in *Vibrio*.

To sum up, although roles of Ig-like domains have been revealed in *Vibrio*, their characterization in *V. parahaemolyticus* has not been established. Therefore, this study aimed to uncover the characteristics and functions of Ig-like proteins and fibrillar adhesins in *V. parahaemolyticus* systematically, giving insights into promising targets for preventing virulence and infection of *V. parahaemolyticus*.

## 2. Results

### 2.1. Distribution of Ig-like Fold Containing Proteins

There were 64, 63, 33 and 42 Pfam (Ig-like fold domains, CL0159) hits identified in *V. parahaemolyticus* RIMD 2210633, *V. parahaemolyticus* CHN25, *V. cholerae* NCTC 9420 and *Escherichia coli* K-12 MG1655 genomes. The heatmap of Pfam hits was generated to present the distributions of multiple Pfams in different genomes (Table 1). Via Table 1, it was indicated that Ig-fold domain distributions were different in four species, the largest divergence was observed in *E. coli*. PF00207, PF02753, PF00630, PF04234, PF06832, PF07703, PF09134, PF10610, PF11806, PF14310, PF16640, PF16967, PF17789, PF17962, PF17970, PF17972, PF17973 and PF18565 (30.3%) were the Pfam domains which only existed in the *E. coli* but not in *Vibrio* species. *E. coli* shared only 1.5% similarity with *V. parahaemolyticus* RIMD 2210633 in Ig-like fold domains, suggesting their different adhesion and signalling mechanisms. *V. cholerae* possesses the least Ig-like fold domains, and only 7.6% of domains were distinctive in this species. *V. parahaemolyticus* RIMD 2210633 and *V. parahaemolyticus* CHN25 shared the most similarity in Ig-like fold domains.

### 2.2. Orthogroup Analysis

OrthoFinder assigned 121 Ig-like containing proteins (91.0% of total) to 40 orthogroups, and 50% of all proteins were in orthogroups with three or more proteins and were contained in the largest 17 orthogroups. There were seven orthogroups present in all genomes, and six of these consisted entirely the single-copy proteins. The results from Orthogroup analysis was in consist with Ig-like fold domain distribution analysis (Table 2), that *E. coli* may have the most different signaling and adhesion mechanism from *V. cholerae* and *V. parahaemolyticus*; meanwhile, *V. parahaemolyticus* RIMD 2210633 and *V. parahaemolyticus* CHN25 may possess the most similar mechanisms of Ig-like fold domains. The single-copy orthologues of these four genomes were OG0000002, OG0000003, OG0000004, OG0000005, OG0000006 and OG0000008.

### 2.3. Identification of Fibrillar Adhesin Like Proteins

Four proteins were identified as fibrillar adhesin-like proteins: WP_005477759.1, WP_005480168.1, WP_005489282.1 and WP_005490731.1. The physico-chemical properties of four fibrillar adhesin-like proteins was analyzed and the results are presented in Table 3. The molecular weight (MW) of fibrillar adhesin-like proteins ranged from 53,630.31 to 638,384.33 Da. The isoelectric point (PI), which depicts the intolerance of surrounding charge, ranged from 3.58 to 4.58. The aliphatic index was used to predict the thermostability, and its results ranged from 70.70 to 92.16. GRAVY (grand average of hydropathicity), depicting the interaction of proteins with water, ranged from −0.097 to −0.464. The stability of proteins was disclosed by the instability index which was below 40, and all four fibrillar adhesin-like proteins were stable.

### 2.4. Three-Dimensional Structure and Protein Interface Analysis

Based on mmseq2 searching algorithm, structural homologues of these four fibrillar adhesin-like proteins have been demonstrated (Table 4). Interestingly, the functions of these homologues were related to adhesion and virulence in other microorganisms, such as *Pseudomonas aeruginosa*, *Azotobacter vinelandii*, *Marinomonas primoryensis*, *V. cholerae* and *Leptospira interrogans*. Most ligands of these homologues were related to CA (calcium ion, Ca^2+^) and ZN (zinc ion, Zn^2+^). The protein interface results indicated promising protein–protein interfaces were present that can be designed as drug targets; residues were coloured according to the Z-score of dL (likelihood difference) score used in BindML tool, from red (predicted protein interfaces, dL Z-scores ≤ −2.0) to grey (predicted other interfaces, dL Z-scores ≤ 0.0) and blue (non-interface).

### 2.5. Molecular Docking

To verify the effects of known virulence inhibitors against *E. coli* [13,14,15,16], interactions between the 277 active compounds and fibrillar adhesins were tested randomly (Supplementary Table S1). Lower binding energy binding affinity (kcal/mol) means stronger binding between the active compounds and the targets, and the top active compounds of each fibrillar adhesins structural model are presented in Table 5.

## 3. Discussion

This study aimed to identify fibrillar adhesins from Ig-like fold-containing proteins in *V. parahaemolyticus*, as well as predict their tertiary structures and binding interfaces. Following this, the phylogenetic ortholog analysis of Ig-like fold containing proteins was examined in a comparative genome manner. Fibrillar adhesins were identified by Pfam domains and HMM search. This study has led to the discovery of four fibrillar adhesins in *V. parahaemolyticus*.

While the domain of PapD_C (Pfam ID: PF02753) generated 10 hits in *E. coli* K-12 MG1655, none of it was present in *V. parahaemolyticus* RIMD 2210633, *V. parahaemolyticus* CHN25, *or V. cholerae* NCTC 9420; this domain is related with pili assembly and attachment mediation to different receptors. *V. parahaemolyticus* and *V. cholerae* were also found to have divergent Ig-like folds, domains of RET_CLD1 (Pfam ID: PF17756), Big_11 (Pfam ID: PF18200), Cadherin (Pfam ID: PF00028) and fn3 (Pfam ID: PF00041) existed only in *V. cholerae*; interestingly these four domains are also related to adhesion or ligand binding functions. The phylogenetically restricted domains and proteins have sparked interest due to the possibility that they may have evolved with distinct binding specificities. The orthogroups analysis assisted in narrowing down the distinctive Ig-like fold proteins in *V. parahaemolyticus*.

Due to the increased binding possibility of target sites to host cells, multiple domains and tandem sequence repeats being contained within a single protein may be adhesins [4,17]. Bacteria express fibrillar adhesins to facilitate attachment onto other microorganisms and/or host cells, and some of them have been reported. For example, CshA interacts with the high-molecular-weight glycoprotein fibronectin (Fn) via an N-terminal non-repetitive region, and it has been proposed that this protein–protein interaction promotes *Streptococcus gordonii* colonization at multiple sites within the host [18]. As a type of surface fibrillar adhesin in *Streptococcus mutans*, SpaP binds to salivary agglutinin glycoprotein and the proline-rich protein of the acquired pellicle on the tooth surface [3]. SraP mediated *Staphylococcus aureus* adhesion to host cells via N-terminal Ig-like domain specifically binding to N-acetylneuraminic acid [19]. However, no information about the identification in *V. parahaemolyticus* has been found. Therefore, this study filled in the knowledge gap and identified four fibrillar adhesin-like proteins, three of which have not been characterized in *V. parahaemolyticus*. Notably, Gram-negative bacteria have been suggested with less fibrillar adhesins as they may use more pili or outer membrane proteins for colonization [20].

Prediction of 3D structures and protein interfaces among structural homologues helped us understand fibrillar adhesin functions in *V. parahaemolyticus*. AprA has been reported as a virulence factor in *P. aeruginosa* which is the homologue of protein WP_005477759.1 [21,22]. The two-domain protein AprA (PDB accession: 1KAP) possesses a calcium-binding parallel beta roll, and unique ligands of CA (calcium ion, Ca^2+^) and ZN (zinc ion, Zn^2+^). Ca^2+^ and Zn^2+^, working as determinants in the host environment, play critical roles in regulating host colonization and bacterial virulence [23]. The predicted results of the protein interface suggested that there were targets that could be employed to design drugs and inactivate the protein. Regarding WP_005489282.1, AlgE6 in *A. vinelandii* and another adhesin in *M. primoryensis* were identified as the homologues while searching against the PDB database. AlgE6 is a calcium-dependent mannuronan C-5 epimerase, and its structure is elongated parallel β-roll with a shallow [12]. The 0.6-μm-long adhesin in *M. primoryensis* was responsible for positioning and gaining access to oxygen and nutrients; the Ig-like fold domain region in this adhesion protein bound with Ca^2+^ and helped the adhesin protein project into medium [24]. WP_005480168.1 is the colonization factor GbpA, acting as an attachment factor in *V. parahaemolyticus*, enabling bacteria to enter seafood, such as prawn (*Macrobrachium rosenbergii*) [25]. LigA and LigB are virulence factors in *Leptospira*, which were identified as homologues to WP_005490731.1; they were involved in adhesion to host cells via extracellular matrix binding and immune evasion [26,27], suggesting similar functions of WP_005490731.1 in *V. parahaemolyticus*. However, proteins of WP_005477759.1, WP_005489282.1 and WP_005490731.1 have not been characterized yet, and they should be verified further in wet labs.

The molecular docking analysis suggested that obacunone, rutin, 8-oxocoptisine and limonin were highly suitable active compounds defending against fibrillar adhesins, revealing applications of these natural plants or activate compounds in the virulence inhibition of *V. parahaemolyticus* with the host. Other natural compounds that are promising resources and have not been examined could be further investigated. This study helped set the foundation of analysis for the identification of virulence inhibition targets and novel active compounds.

Ig-like fold proteins have been shown to be effective in regulating *V. parahaemolyticus* and other veterinary pathogens. Genetic regulators or repressors have been proposed to defend against biofilms, however, there exists a three-dimensional matrix outside bacterial communities which has high tolerance against disinfectants; the chemicals in disinfectants would be difficult to diffuse into the gel like matrix and attack the bacteria. The Ig-like domain was shown to have carbohydrate (lectin)-binding activity, acting as a surface glycan; therefore, it was used to develop methods for detecting *E. coli* biofilms with recombinant antibodies, thereby locating and destroying the biofilm matrix [28]. Antimicrobial resistance can also be lowered by Ig-like domains. For example, the expression of RSP, a protein which contains an Ig-like domain, is required in the conjugative transfer of IncHI plasmids in antimicrobial resistant *Salmonella* isolates [29]. Ig-like domains can assist in understanding *Vibrio* phage and help develop or modify phage cell surface proteins to easily bind *V. parahaemolyticus*. The identification of fibrillar adhesins in *V. parahaemolyticus* help identify those in other pathogens, which therefore will prevent pathogen colonization, quorum sensing and virulence expansion.

## 4. Conclusions

This study examined Ig-like-folded proteins and novel fibrillar adhesin-like proteins in *V. parahaemolyticus*. The results underline characteristics and unveil the orthologous relationship of Ig-like folded proteins in *V. parahaemolyticus*. There were three novel fibrillar adhesin-like proteins that have been identified in *V. parahaemolyticus* within this study. The molecular docking analysis set a foundation for discovering adhesion sites of virulence inhibitors and paved the way for finding out prior drugs for targeting cell attachment and virulence expansion for *V. parahaemolyticus* and other pathogens.

## 5. Materials and Methods

### 5.1. Definition of Profiles for Ig-like Fold Proteins

Ig proteins were defined based on Pfam database across *V. parahaemolyticus* RIMD 2210633, *V. parahaemolyticus* CHN25, *V. cholerae* NCTC 9420 and *E. coli* K-12 MG1655. Ig fold domains in bacteria were identified which belong to CL0159, the Ig-like fold superfamily [30]. The selection of proteins was using HMMER filter parameters as follows: (1) sequence search with e-value below 1 × 10^−5^; (2) searching a domain score of above 200; (3) the protein sequence identity of over 70%; (4) predicted domains with more than 15 amino acids. Sequence hits were summarized from HMMER searching results.

### 5.2. Orthogroup Inference of Ig-Fold Containing Proteins

Amino acid sequences of Ig-fold containing proteins were examined for orthogroup clustering via OrthoFinder using DIAMOND as the all-versus-all alignment tool. This examination of homologues was based on the MCL algorithm.

### 5.3. Identification and Characterization of Fibrillar Adhesin-like Proteins

Proteins that met standards as follows were considered as the fibrillar adhesin-like proteins [30,31]: (1) existence of tandem repeats; (2) proteins that accommodate Pfam domains belonged to clan CL0159; (3) proteins that were identified as virulence factors and the sublocation was at the extracellular location.

The subcellular localization of fibrillar adhesin-like proteins was predicted using CELLO2GO (http://cello.life.nctu.edu.tw/cello2go/, accessed on 2 December 2021), which is a web platform to describe gene ontology (GO)-type categories and subcellular localizations based on machine-learning algorithm and BLAST homology searching approaches. The virulence of fibrillar adhesin-like proteins was predicted using VirulencePred (http://203.92.44.117/virulent/index.html, accessed on 2 December 2021) that applied a bi-layer cascade support vector machine (SVM) as the algorithm method. The theoretical physico-chemical properties were examined via ProtParam tool available at ExPASy server.

### 5.4. Structure Prediction and Evaluation of Fibrillar Adhesin-like Proteins

Amino acid sequences of the promising fibrillar adhesin-like proteins were loaded into the PDB database, which aims to predict 3D protein structures and biological functions and obtains their structural homologues. The protein interface prediction was performed via BindML (https://kiharalab.org/bindml/plus/, accessed on 2 December 2021).

### 5.5. Molecular Docking of Virulence Inhibitors with Fibrillar Adhesion Proteins

The chemical compounds of virulence inhibitors against *E. coli* were collected from SymMap (http://symmap/org/, accessed on 3 February 2022), which is an integrated traditional Chinese medicine (TCM) database, and the sdf database was generated via PubChem (https://pubchem.ncbi.nlm.nih.gov/, accessed on 3 February 2022) via PubChem ID. The chemical compounds were decorated by removing the ligands and water motifs, reviewing this and optimizing the mutation sites, and adding hydrogen through the Pymol 2.3 and UCSF Chimera 1.14rc software. 3D structural information of these four identified fibrillar adhesin proteins used the best-fit molecules based on the PDB database. The binding ability sites, interaction between compounds and fibrillar adhesins were performed using AutoDock vina (https://vina.scripps.edu/, accessed on 3 February 2022).

## Figures and Tables

**Table 1:** Ig-like fold domain distribution heatmap of *E. coli*, *V. cholerae* and *V. parahaemolyticus*.

Pfam ID	Pfam	*Escherichia coli* K-12 MG1655	*Vibrio cholerae* NCTC 9420	*Vibrio parahaemolyticus* CHN25	*Vibrio parahaemolyticus* RIMD 2210633
PF00028	Cadherin	0	1	0	0
PF00041	fn3	0	1	0	0
PF00207	A2M	1	0	0	0
PF00630	Filamin	1	0	0	0
PF00703	Glyco_hydro_2	3	1	2	2
PF00801	PKD	0	1	1	1
PF00932	LTD	0	1	1	1
PF01345	DUF11	0	0	1	1
PF01835	MG2	1	0	0	1
PF02010	REJ	0	0	1	1
PF02368	Big_2	0	1	1	1
PF02369	Big_1	1	0	0	0
PF02753	PapD_C	10	0	0	0
PF02903	Alpha-amylase_N	1	0	1	1
PF02927	CelD_N	0	1	1	1
PF03067	LPMO_10	0	2	2	2
PF03160	Calx-beta	0	1	2	1
PF03174	CHB_HEX_C	0	1	1	1
PF03404	Mo-co_dimer	0	0	1	1
PF04234	CopC	1	0	0	0
PF04379	DUF525	1	1	1	1
PF05345	He_PIG	0	0	1	1
PF05753	TRAP_beta	0	0	1	1
PF06832	BiPBP_C	1	0	0	0
PF07233	DUF1425	1	1	1	1
PF07495	Y_Y_Y	0	1	0	0
PF07703	A2M_BRD	1	0	0	0
PF08329	ChitinaseA_N	0	1	1	1
PF09134	Invasin_D3	1	0	0	0
PF09619	YscW	1	1	2	2
PF10029	DUF2271	0	0	1	1
PF10610	Tafi-CsgC	1	0	0	0
PF10633	NPCBM_assoc	0	0	2	2
PF11412	DsbC	1	1	2	2
PF11614	FixG_C	0	1	1	1
PF11806	Enterochelin_N	1	0	0	0
PF11940	DUF3458	1	1	1	1
PF11974	bMG3	1	0	0	0
PF12262	Lipase_bact_N	0	1	1	1
PF13584	BatD	0	1	2	2
PF13629	T2SS-T3SS_pil_N	0	0	2	2
PF13860	FlgD_ig	1	1	3	3
PF14310	Fn3-like	1	0	0	0
PF14467	DUF4426	0	1	1	1
PF16184	Cadherin_3	0	0	1	1
PF16353	LacZ_4	2	1	1	1
PF16640	Big_3_5	1	0	0	0
PF16655	PhoD_N	0	0	1	1
PF16967	TcfC	1	0	0	0
PF17753	Ig_mannosidase	0	0	1	1
PF17756	RET_CLD1	0	1	0	0
PF17786	Mannosidase_ig	0	0	1	1
PF17789	MG4	1	0	0	0
PF17803	Cadherin_4	0	1	4	4
PF17892	Cadherin_5	0	1	5	5
PF17957	Big_7	0	2	2	2
PF17962	bMG6	1	0	0	0
PF17963	Big_9	0	1	5	5
PF17967	Pullulanase_N2	0	0	1	1
PF17970	bMG1	1	0	0	0
PF17972	bMG5	1	0	0	0
PF17973	bMG10	1	0	0	0
PF18200	Big_11	0	1	0	0
PF18565	Glyco_hydro2_C5	1	0	0	0
PF18911	PKD_4	0	2	2	3
PF19076	CshA_repeat	0	0	1	1

Different colors in this table presented various abundance of pfam superfamilies, yellow to green indicates the increasing abundance, the number in each cell reflected the pfam superfamily numbers existed in each bacteria strain.

**Table 2:** Orthogroup analysis of Ig-like fold containing proteins in *E. coli*, *V. cholerae* and *V. parahaemolyticus*.

Orthogroup	*V. parahaemolyticus* RIMD 2210633	*V. parahaemolyticus* CHN25	*E. coli* K-12 MG1655	*V. cholerae* NCTC 9420
OG0000000			NP_414682.1, NP_415064.1, NP_415245.1, NP_415459.1, NP_415464.4, NP_416613.1, NP_416839.1, NP_417519.4, NP_417612.1, NP_418736.3	
OG0000001	WP_005482309.1	WP_065870880.1	NP_414878.1, NP_416134.1, YP_026199.1	WP_001243585.1
OG0000002	WP_005456243.1	WP_005456243.1	NP_415622.1	WP_001261381.1
OG0000003	WP_005459620.1	WP_005459620.1	NP_414592.1	WP_000383338.1
OG0000004	WP_005461146.1	WP_005461146.1	NP_414987.3	WP_000756880.1
OG0000005	WP_005462292.1	WP_005462292.1	NP_415593.1	WP_000929365.1
OG0000006	WP_005478597.1	WP_017449375.1	NP_418559.1	WP_001259538.1
OG0000007	WP_005480222.1, WP_005480358.1	WP_065871343.1, WP_205390631.1		
OG0000008	WP_005481930.1	WP_065870668.1	NP_415452.1	WP_071919720.1
OG0000009	WP_005455369.1	WP_065871306.1		WP_000815658.1
OG0000010	WP_005461703.1	WP_005461703.1		WP_001233676.1
OG0000011	WP_005478358.1	WP_162780945.1		WP_000238825.1
OG0000012	WP_005479039.1	WP_065870865.1		WP_071919742.1
OG0000013	WP_005479087.1	WP_065870903.1		WP_000925616.1
OG0000014	WP_005479129.1	WP_025504563.1		WP_071919697.1
OG0000015	WP_005480168.1	WP_005480168.1		WP_000744635.1
OG0000016	WP_005480704.1	WP_065870579.1		WP_000848953.1
OG0000017	WP_005481163.1	WP_015297519.1		WP_000076153.1
OG0000018	WP_005482420.1	WP_065870820.1	NP_414937.2	
OG0000019	WP_005482861.1	WP_065871072.1		WP_001894571.1
OG0000020	WP_005482868.1	WP_065871083.1		WP_000873599.1
OG0000021	WP_005488937.1	WP_065870960.1		WP_000426028.1
OG0000022	WP_005489828.1	WP_065870398.1		WP_000914817.1
OG0000023	WP_011105887.1	WP_065870696.1		WP_080488946.1
OG0000024	WP_005454128.1	WP_005454128.1		
OG0000025	WP_005456668.1	WP_065870884.1		
OG0000026	WP_005462598.1	WP_065870556.1		
OG0000027	WP_005463605.1	WP_065871223.1		
OG0000028	WP_005463939.1	WP_005463939.1		
OG0000029	WP_005477556.1	WP_079879954.1		
OG0000030	WP_005477759.1	WP_065870697.1		
OG0000031	WP_005479027.1	WP_065870427.1		
OG0000032	WP_005479631.1	WP_005479631.1		
OG0000033	WP_005479868.1	WP_065871168.1		
OG0000034	WP_005481629.1	WP_065870795.1		
OG0000035	WP_005482097.1	WP_065871538.1		
OG0000036	WP_005489282.1	WP_065871245.1		
OG0000037	WP_005490731.1	WP_205390630.1		
OG0000038	WP_005492007.1	WP_205390635.1		
OG0000039	WP_011105908.1	WP_065870717.1		

The analysis result was generated from OrthoFinder automatically, protein(s) presented in each cell was/were named using NCBI reference accession(s).

**Table 3:** Physico-chemical properties of fibrillar adhesin-like proteins.

Protein Accession	Molecular Weight (MW)	Isoelectric Point (PI)	Grand Average of Hydropathicity (GRAVY)	Instability Index	Aliphatic Index
WP_005477759.1	338,024.95	3.72	−0.242	19.69	87.55
WP_005480168.1	53,630.31	4.58	−0.464	29.84	70.70
WP_005489282.1	638,384.33	3.58	−0.097	19.27	92.16
WP_005490731.1	75,993.93	4.5	−0.27	36.66	88.21

**Table 4:** Prediction of 3D structure and protein interfaces.

Protein Accession	Homologues						
	3D Structural Models	PDB ID	Organism	Protein Annotation	Unique Ligands	Chains	Protein Interface
WP_005477759.1	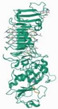	1KAP	*Pseudomonas aeruginosa*	A two-domain protein AprA with a calcium binding parallel beta roll motif	ZN, CA	A [auth P]	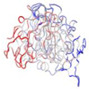
						B [auth I]	
	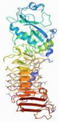	1AKL	*P. aeruginosa*	Alkaline protease	ZN, CA	A	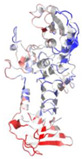
	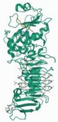	3VI1	*P. aeruginosa*	Alkaline protease complexed with Substance P (1–6)	ZN, CA	C, D	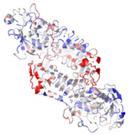
						A, B	
WP_005489282.1	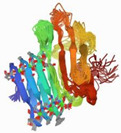	2ML1	*Azotobacter vinelandii*	AlgE6R1 subunit from the *Azotobacter vinelandii* Mannuronan C5-epimerase AlgE6	CA	A	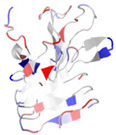
	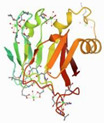	5JUH	*Marinomonas primoryensis*	C-terminal domain [12] of MpAFP, a 1.5-MDa adhesin that binds its Antarctic bacterium to diatoms and ice	CA	A	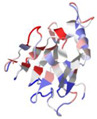
WP_005480168.1	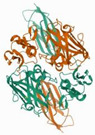	2XWX	*V. cholerae*	Colonization factor GbpA	-	A, B	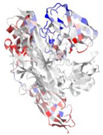
WP_005490731.1	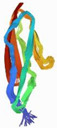	2N7S	*Leptospira interrogans*	Leptospiral immunoglobulin-like protein A (LigA), involved in the interaction of pathogenic *Leptospira* with mammalian host	-	A	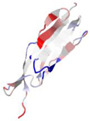
	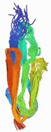	2MH4	*L. interrogans*	LigB-like protein	-	A	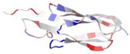

**Table 5:** Promising activate compounds and their binding affinity analysis targeting fibrillar adhesins.

WP_005477759.1 (1KAP)			WP_005489282.1 (2ML1)		WP_005480168.1 (2XWX)		WP_005490731.1 (2N7S)	
Ligand		Binding Affinity	Ligand	Binding Affinity	Ligand	Binding Affinity	Ligand	Binding Affinity
1	Obacunone	−9.2	Spinasterol	−8.5	Cardenolide glycoside	−6.9	Limonin	−9.9
2	Pycnamine	−9.1	Stigmasterin	−8.4	Pycnamine	−6.9	8-Oxocoptisine	−9.7
3	Rutin	−9.1	Higenamine	−8.3	8-Oxocoptisine	−6.9	Obacunone	−9.5
4	Coptisine	−8.8	Obacunone	−8.3	Rutin	−6.8	Ursolic Acid	−9.5
5	Limonin	−8.8	Sitosterol	−8.3	Coptisine	−6.8	Palmidin A	−9.3

## Data Availability

Genome data is publicly available in NCBI database (https://www.ncbi.nlm.nih.gov/), other data in this study is contained within the article and Supplemental Table S1.

## Supplementary Materials

The following supporting information can be downloaded at: https://www.mdpi.com/article/10.3390/toxins14020133/s1, Table S1: Examined compounds and PubChem annotation.
